# A New Solid-Phase Immunosorbent for Selective Binding of Desmoglein 3 Autoantibodies in Patients with Pemphigus Vulgaris

**DOI:** 10.32607/actanaturae.10893

**Published:** 2020

**Authors:** T. V. Abramova, M. V. Spilevaya, A. A. Kubanov

**Affiliations:** State Research Center of Dermatovenereology and Cosmetology, Ministry of Healthcare of the Russian Federation, Moscow, 107076 Russia

**Keywords:** autoantibodies, acantholysis, desmoglein, pemphigus, treatment, immunosorbent

## Abstract

Autoantibodies, immunoglobulins G (IgG) against the desmosomal proteins desmogleins 1 and 3, play a significant role in the pathogenesis of pemphigus vulgaris. The basic therapy for pemfigus includes systemic corticosteroids, but their use should be as brief as possible because of the severe side effects. In cases of corticosteroid- resistant pemfigus, adjuvant therapy, in particular extracorporeal methods, is used. The most effective and safest extracorporeal therapy is immunosorbtion. Immunosorbtion is based on the removal of pemphigus antibodies from the blood using an affinity sorbent during a therapeutic apheresis procedure. Existing immunosorbents are nonselective and increase the risk of infection. We designed an immunosorbent based on an agarose matrix, Affi-Gel 15, and human recombinant desmoglein 3, as a ligand, for a selective removal of autoantibodies from pemphigus patients’ sera. It was shown on a pemphigus experimental model *in vivo *(neonatal Balb/c mouse model) and *in vitro *that the immunosorbent can effectively remove desmoglein 3-associated autoantibodies. The experimental results demonstrate that the solid-phase matrix immunosorbent Affi-Gel 15–Dsg3 is a promising product for the development of pemphigus therapy.

## INTRODUCTION

To date, there are several dozen known autoimmune diseases the pathogenesis of which is determined mainly by the response of the immune system to autoantigens. One of the severe autoimmune bullous diseases is pemphigus, which affects the skin and/or mucous membranes. The main antigens of autoaggression in pemphigus are transmembrane desmosomal glycoproteins – desmogleins 3 and 1 (Dsg3 and Dsg1) [1, 2, 3]. Pemphigus patients produce autoantibodies, immunoglobulins G (IgGs) that exhibit high tissue specificity and affinity for appropriate antigens. The binding of humoral autoantibodies to the extracellular domain of cell adhesion proteins leads to damage to proteins by proteolytic enzymes and to disruption of the connections that bind adjacent epithelial cells: acantholysis [4, 5]. There are different clinical forms of pemphigus: vulgaris and foliaceus [6, 7, 8]. Anti-Dsg3 autoantibodies are detected in the blood serum of 80–100% of patients with pemphigus vulgaris [9, 10], the presence of which is considered a key factor that promotes the development of the clinical phenotype of pemphigus vulgaris [11].

Pemphigus treatment consists in suppressing the synthesis and elimination of autoantibodies to keratinocyte proteins. The baseline medications for pemphigus are systemic glucocorticosteroids, the prolonged use of which may cause severe complications that aggravate the condition of patients and worsen the prognosis of the disease [12, 13]. The issue of therapy in severe and steroid-resistant forms of the disease remains topical. To reduce high doses of systemic GCS, adjuvant therapy (cytostatics, extracorporeal methods) is prescribed. Various combined methods, in particular a combination of glucocorticosteroid drugs and cytostatics, can somewhat reduce the course dose of hormonal drugs; however, the degree of complications still remains high; in addition, immunosuppressive agents aggravate the existing immunodeficiency state of patients [7, 8, 14, 15].

In recent decades, attempts have been made to treat pemphigus with extracorporeal therapies, such as hemosorption, plasmapheresis, and immunosorption. For immunosorption, three types of sorbents are used: non-selective, low-selective, and high-selective. Nonselective sorbents (dextransulfate, tryptophan, phenylalanine- based sorbents, etc.) are capable of sorbing plasma components, such as fibrinogen, albumin, lipids, and immunoglobulins. Low-selective sorbents (with an immobilized staphylococcal protein A) have affinity for a certain fraction of plasma proteins. However, the use of non-selective and low-selective immunosorbents eliminates not only pathogenically significant autoantibodies, but also other immunoglobulins and the immune complexes necessary for a normal functioning of the immune system, which limits their application [16-21].

The most effective and safe treatments for autoimmune diseases include an extracorporeal method that uses specific high-selective immunosorbents that extract only certain proteins without affecting the concentrations of other components of a patient’s plasma [21].

The purpose of this study was to develop a solidphase immunosorbent for the targeted elimination of desmoglein 3 antibodies from the blood serum of patients with pemphigus vulgaris and to evaluate the effectiveness of this sorbent.

## EXPERIMENTAL

**Patients**

Peripheral blood samples were obtained from 22 patients with pemphigus vulgaris (main group) with activity of 200 or more RU/ml and from 14 healthy individuals (control group). The diagnosis of pemphigus was established on the basis of a clinical examination and results of laboratory tests (cytological, pathomorphological, and immunohistochemical (indirect immunofluorescence assay (IFA) using *ex vivo *confocal laser scanning microscopy (CLSM)). All patients provided written informed consent in order to participate in the study. The study was performed in compliance with applicable legal and ethical standards.

**Enzyme immunoassay for determining desmoglein 3 antibodies**

The levels of desmoglein 3 antibodies in the blood serum of patients with pemphigus vulgaris and healthy individuals were determined by an enzyme immunoassay (EIA) using Anti-Desmoglein 3 ELISA (IgG) test systems (Euroimmun, Canada). Activity was determined in relative units per mL of serum (RU/mL).

**Isolation of IgG antibodies**

IgG antibodies were isolated from a pool of blood sera of pemphigus patients and healthy individuals by affinity chromatography using protein G sepharose (Bialexa, Russia). Protein purity was evaluated by denaturing polyacrylamide gel electrophoresis [22]. Immunoglobulins were dialyzed against pH 7.3 phosphate buffer and concentrated by ultrafiltration in Amicon filter units (Millipore, France). The activity of Dsg3 antibodies was determined using an Anti-Desmoglein 3 ELISA (IgG) test kit. The IgG concentration was measured using an IgG total-EIA-BEST kit (Vector-Best, Russia). Isolated proteins were stored at –20°C.

**Preparation of an affinity sorbent for the binding of Dsg3 antibodies**

The affinity sorbent was prepared based on the Affi- Gel 15 agarose matrix (Bio-Rad, USA) and recombinant human Dsg3 produced in a *Saccharomyces cerevisiae *yeast culture [23] (MyBiosource, USA).

Dsg3 was immobilized on the Affi-Gel 15 matrix according to the procedure from the gel manufacturer [24]. The process of protein binding to the matrix was monitored by electrophoresis under denaturing conditions. Remaining free Affi-Gel 15 ester groups were blocked by adding 0.1 mL of 1 M ethanolamine HCl (pH 8.0) per 1 mL of gel.

The sorption process was monitored using a Affi-Gel 15 matrix that was similarly treated, but without the addition of Dsg3.

**Evaluation of adsorption capacity**

An Eppendorf tube containing 20 μL (VC) of the sorbent equilibrated with loading buffer (20 mM phosphate-saline buffer, pH 7.4) was added to 100 μL (VIgG) of the IgG fraction with a known activity (C0) from sera of pemphigus patients. The suspension was incubated at room temperature and stirring (2 rpm) for 30 min, followed by precipitation of the sorbent by centrifugation (3,000 rpm, 1 min). In the supernatant, the activity of the material after sorption (CP) was determined by EIA. The capacity of the carrier (A) was calculated using the formula:

A = (C_0_ – C_P_) × V_IgG_/V_C_ (1).

**Sorbent regeneration**

After each immunoadsorption procedure, the sorbent was regenerated with a 0.05 M glycine buffer (pH 2.5). Some 20 μL of the sorbent was added with 100 μL of buffer and incubated at room temperature and stirring at 4 rpm for 15 min, followed by precipitation of the sorbent by centrifugation at 3000 rpm for 1 min. Glycine buffer was washed out three times with load buffer (20 mM phosphate-buffered saline, pH 7.4).

**Evaluation of sorbent stability during regeneration**

We studied the effect of regeneration on the sorption properties of the Affi-Gel 15–Dsg3 immunosorbent. Some 100 μL of the serum with a level of activity of 200 RU/mL from a pemphigus patient was added to 20 μl of the sorbent. Suspensions were incubated at room temperature and stirring at 2 rpm for 30 min and precipitated by centrifugation (3 000 rpm, 1 min), the supernatant was removed, the sorbent was regenerated according to the described procedure, and a fresh portion of serum was added. The procedure was repeated 12 times, with residual serum activity being determined in the supernatant after each procedure.

**Preparations for administration to laboratory animals**

Pemphigus was induced in laboratory animals *in vivo* using IgG isolated from a pool of sera from pemphigus vulgaris patients with anti-Dsg3 antibody activity of 12 000 RU/mL.

To prevent the pathogenic effect of anti-desmoglein antibodies *in vivo*, 1 mL of IgG with activity of 12 000 RU/mL was adsorbed onto 500 μL of the synthesized immunosorbent (at room temperature and stirring at 2 rpm for 30 min), followed by precipitation of the sorbent by centrifugation (3 000 rpm, 1 min). Activity of the preparation after sorption was determined in the supernatant.

The animals of the control group were injected with IgG isolated from a pool of blood sera from healthy individuals, as well as with sterile phosphate-buffered saline pH 7.3.

Before administration to laboratory animals, all IgG solutions were sterilized by filtration through Millex filters (Merck Millipore, USA) with a pore size of 0.22 μm.

**Experiments on laboratory animals**

The experiments were performed in the Laboratory of Biological Testing of the Branch of the Shemyakin and Ovchinnikov Institute of Bioorganic Chemistry – Pushchino Nursery for Laboratory Animals (Russia, Pushchino). The nursery has AAALAC international accreditation. The system for quality control of laboratory animals production in the Nursery is certified to international requirements of ISO 9001:2008. All the studies on the animals were performed in accordance with the Good Laboratory Practice Rules in the Russian Federation (Order of the Ministry of Health of the Russian Federation of April 01, 2016 No. 199n “On Approval of the Rules for Good Laboratory Practice”; GOST 53434–009 Principles of Good Laboratory Practice (updated March 01, 2018); Resolution of the Local Ethics Committee for Biomedical Research, Branch of the Shemyakin and Ovchinnikov Institute of Bioorganic Chemistry of the Russian Academy of Sciences (No. 137/17 of December 27, 2017)).

Given a similar distribution pattern of desmogleins that are the main antigens in neonatal mice and humans in pemphigus [25], neonatal inbred BALB/c male mice under 24 h of age with a specified pathogen-free (SPF) status were selected for the generation of an experimental model and subsequent evaluation of the effectiveness of Dsg3 autoantibody sorption *in vivo*.

A series of experiments included the following: clinical observation with identification of erosions and/or blisters on the skin of mice; sampling of autopsy material (skin biopsy samples) from mice for subsequent morphological (detection of acantholysis) and immunohistochemical (identification of fixed IgG in the skin) studies.

**Morphological study**

In a morphological study, 5-μm skin biopsy sections were stained with hematoxylin-eosin in a Leica Autostainer XL ST5010 device (Germany). The resulting histological preparations were examined using a Leica DM4000B light microscope (Germany).

**Immunohistochemical study**

For the immunohistochemical study (indirect immunofluorescence assay (IFA)), sections were treated with primary antibodies (rabbit anti-human IgG polyclonal antibodies (Cell Marque antibody, USA)), incubated at room temperature for 1 h, and washed (3 times for 5 min each) in phosphate-buffered saline supplemented with Tween-20. At the next stage, the sections were treated with goat anti-rabbit IgG secondary antibodies (Epitomics, USA) labeled with a fluorochrome (Alexa Fluor 488), incubated at room temperature for 1 h, washed as described above, dried, placed in a medium containing a nucleotide-specific fluorochrome (4,6-diamidino- 2-phenylindole, DAPI) (Alexa Fluor 405), and covered with a coverslip.

## RESULTS AND DISCUSSION

**IgG isolation**

Two IgG fractions were obtained using the described methods: from a pool of blood sera with activity of 15 000 RU/mL from pemphigus patients and from a pool of blood sera with activity of less than 10 RU/mL from healthy individuals. The IgG concentration in both fractions was 140 mg/mL.

**Preparation and evaluation of the effectiveness of the affinity sorbent for the removal of Dsg3 antibodies**

**Fig. 1:**
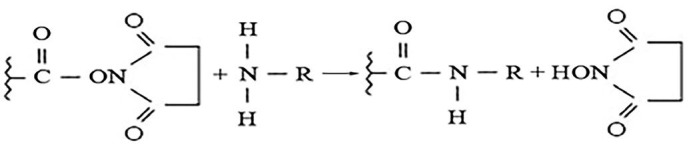
Schematic of the coupling of Affi-Gel 15 for the primary amino group of a protein

Affi-Gel 15 is an agarose and polyacrylamide gel with 15-membered hydrophilic spacers modified by the addition of N-hydroxysuccinimide. Affi-Gel 15 is the complementary affinity medium for rapid and high-efficiency coupling for primary amino group ligands (*Fig. 1*).

Affi-Gel 15 forms a stable amide bond with the primary amino groups of proteins with an isoelectric point (pI) of ≤ 6.5. One of these proteins is recombinant human desmoglein 3 (Dsg3) with pI = 5.7. Dsg3 is immobilized on an affinity carrier via the primary amino groups of the protein.

**Fig. 2:**
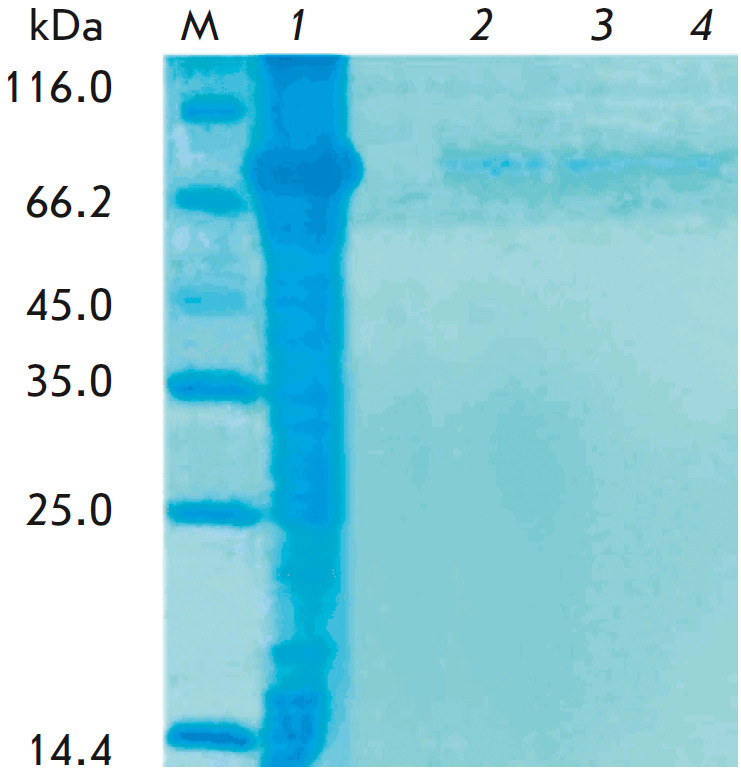
Electropherogram of recombinant human Dsg3 (MW 77 kDa) before (*lane 1*) and after (*lanes 2–4*) immobilization on Affi-Gel 15

Immobilization of Dsg3 was controlled by electrophoresis based on the difference between an added amount of the protein and an amount of the residual protein in the first three volumes of the wash buffer from the sorbent after the immobilization procedure (*Fig. 2*).

The presence of Dsg3 traces in the wash buffer indicates that most of the protein is bound to the agarose matrix.

**Table 1:** Structures of K_V_-channels alone and in complex with charybdotoxin used in homology modeling studies

Baseline serum activity C_0_, RU/mL	Affi-Gel 15–Dsg3		Affi-Gel 15	
	Equilibrium activity C_P_, RU/mL	Adsorption capacity A, RU/mL	Equilibrium activity C_P_, RU/mL	Adsorption capacity A, RU/mL
40	20	100	100	50
120	20	500	500	350
320	40	1,400	1,400	400
400	40	1,800	1,800	400
480	80	2,000	2,000	375
800	100	3,500	3,500	450
1,000	300	3,500	4,000	400
1,100	400	3,500	3,500	425

Thus, we developed an immunosorbent based on an agarose matrix and recombinant human Dsg3, as a ligand, for the selective removal of autoantibodies from the blood sera of patients with pemphigus vulgaris [26].

**Evaluation of the adsorption capacity**

To determine the capacity of the synthesized affinity sorbent and an unmodified Affi-Gel 15 matrix for anti-desmoglein IgG, as well as to generate an experimental adsorption isotherm, eight individual blood sera with a baseline anti-Dsg3 antibody activity in a range of 40–11,000 RU/mL from pemphigus patients were tested using affinity chromatography, followed by determination of the adsorption capacity according to formula (1) (*Table 1*).

**Fig. 3:**
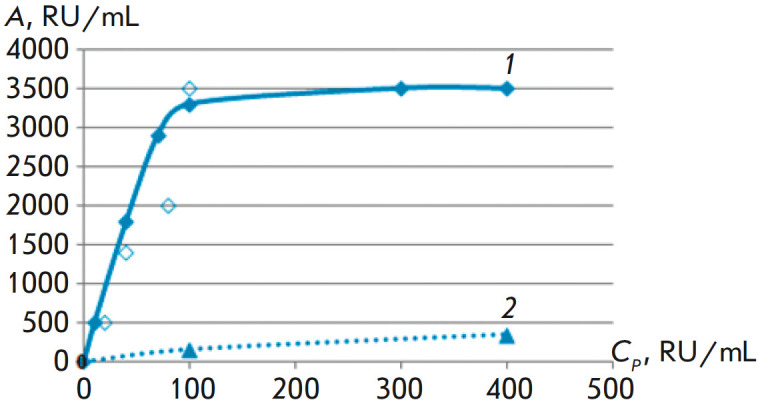
Sorption isotherms of anti-desmoglein IgG from a pool of blood sera from patients with pemphigus:* 1 *– Affi-Gel 15–Ds

The experimental sorption isotherm, which reflects the dependence of adsorption on the equilibrium activity of the serum, shows that the sorption capacity of the selective immunosorbent is 3 500 RU/mL and significantly exceeds that of the unmodified. developed matrix (400–450 RU/mL) (*Fig. 3*).

**Table 2:** Assessment of the severity of pemphigus signs in experimental animals

Technique/Group	Clinical picture	Morphological study	Immunohistochemical study (IIF)
A	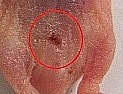 Erosion in the abdominal region	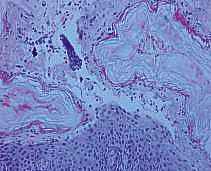 Supra-epidermal acantholysis (hematoxylineosin staining, × 200)	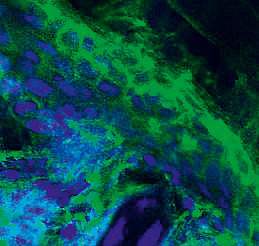 IgG fixation in the intercellular spaces of the epidermis (× 20)
B	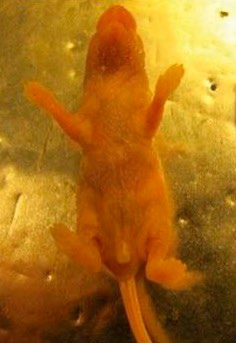 No rashes on the skin.	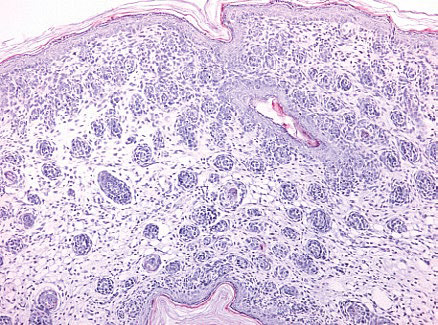 The epidermis is unchanged, with well-defined layers, there are no acantholysis signs. The dermis is unchanged; there are weak inflammation signs represented by slight lymphohistiocytic infiltration in the deep dermis layers (stained with hematoxylin-eosin, × 200).	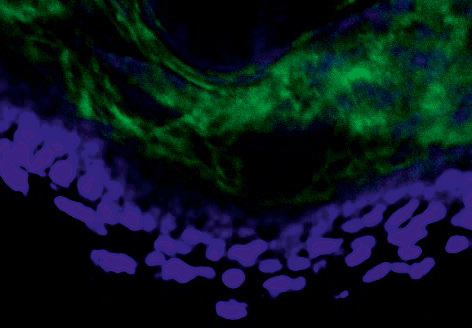 There is no distinctive fixation of IgG deposits in the epidermis. Slight diffuse IgG infiltration is detected in the upper dermis (× 20).

Thus, we experimentally demonstrated a high sorption capacity for the developed immunosorbent for the binding of human anti-Dsg3 autoantibodies from the blood sera of patients with pemphigus vulgaris.

**Investigation of sorbent stability during regeneration**

**Fig. 4:**
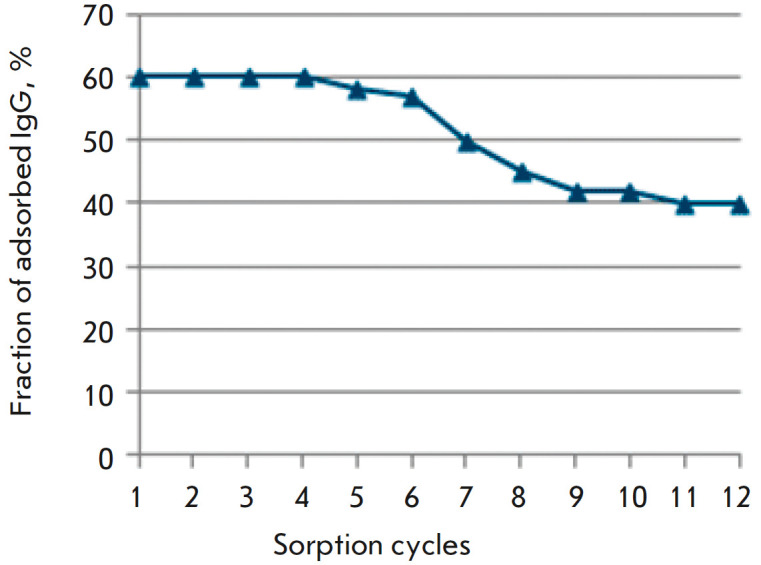
Changes in the sorption activity of the Affi-Gel 15–Dsg3 immunosorbent during 12 chromatography cycles with intermediate regeneration

We performed 12 chromatography cycles of blood serum with activity of 200 RU/mL from a pemphigus patient with intermediate regeneration. The first six cycles did not reveal a change in the sorption characteristics of the synthesized immunosorbent. In the course of the next six cycles, the sorption ability decreased from 60 to 40% (*Fig. 4*).

Therefore, we had experimentally demonstrated the stability of the synthesized immunosorbent and its suitability for multiple use.

**Evaluation of the effectiveness of the selective immunosorbent *in vivo***

To determine the effectiveness of immunoadsorption, we compared the development of pemphigus symptoms in laboratory animals that were injected with the IgG fraction from a pool of patient blood sera (anti- Dsg3 antibody activity of 12 000 RU/mL) and the same preparation after chromatography on the synthesized immunosorbent. The residual activity after chromatography was 2 600 RU/mL. The following preparations were used in in vivo experiments: No. 1 – IgG with activity of 15 000 RU/mL; No. 2 – IgG with activity of 2 600 RU/mL after interaction with the immunosorbent; No. 3 – IgG isolated from a pool of blood sera from healthy individuals [27, 28].

The preparations were administered intraperitoneally to four groups of mice (10 animals each) in 30 μL, twice, with an interval of 24 h: group A –preparation No. 1; group B –preparation No. 2; group C –preparation No. 3; group D –sterile phosphate-saline buffer. Groups C and D were considered as controls.

The development of pemphigus symptoms (clinical, morphological, immunohistochemical) in all animal groups was evaluated within 48 h after the last injection.

Group A mice injected with preparation No. 1 developed single erosions in the abdominal region and positive Nikolsky’s symptom. A morphological study of autopsy material from the mouse skin revealed a pathognomonic sign of pemphigus –suprabasal acantholysis. An immunohistochemical study of mouse skin cryosections in this group revealed pronounced IgG fixation in the intercellular spaces of the epidermis over a long distance, with the formation of a distinctive network structure (the mean luminescence intensity of IgG was 1,008.6 relative units) (*Table 2*).

In group B, mice injected with preparation No. 2 obtained from pemphigus patients, after interaction with the Affi-Gel 15–Dsg3 immunosorbent, did not develop clinical or morphological signs of pemphigus. Examination of mouse autopsy material by IFA revealed diffuse IgG fixation in the intercellular spaces of the suprabasal epidermal layers, without the formation of a distinctive network structure (a luminescence intensity of 380.5 relative units) (*Table 2*).

In the control groups of laboratory animals (C, D), there were no clinical, morphological signs of pemphigus. An immunohistochemical study of the autopsy material of mice did not reveal IgG fixation in the epidermis.

Therefore, *in vivo *experiments confirmed the effectiveness of the Affi-Gel 15–Dsg3 selective immunosorbent in reducing the activity of autoantibodies in the blood sera of pemphigus patients. The use of the immunosorbent decreased the level of Dsg3 antibodies in the blood serum samples of pemphigus patients and prevented the development of clinical, pathomorphological, and immunohistochemical signs of the disease in laboratory animals.

## CONCLUSION

We used the Affi-Gel 15 agarose matrix and recombinant human Dsg3, as a ligand, to synthesize a Affi- Gel 15–Dsg3 immunosorbent that enables selective removal of antibodies (IgG) from a desmosomal structural component (desmoglein 3) that plays a pathogenic role in the development of pemphigus vulgaris. The sorption capacity of the developed immunosorbent is 3 500 RU/mL, with an unmodified Affi-Gel 15 matrix capacity not exceeding 450 RU/mL. The developed immunosorbent is characterized by stability, which makes it suitable for multiple uses. The clinical effectiveness of the sorbent was confirmed in *in vivo *experiments: desmoglein 3 autoreactive antibodies subjected to chromatography on Affi-Gel 15–Dsg3 lose their ability to induce clinical, morphological, and immunohistochemical signs of pemphigus in laboratory animals. Therefore, the synthesized selective immunosorbent Affi-Gel 15–Dsg3 has a combination of properties that make it promising for the development of solid-phase matrices for the treatment of patients with pemphigus vulgaris.

## Abbreviations

EIA: enzyme immunoassay
Dsg3: desmoglein 3
IgG: immunoglobulin G
Affi-Gel: gel for affinity chromatography
BALB/c (Bagg albino C): inbred line of white mice
GCS: glucocorticosteroid
IFA: indirect immunofluorescence assay
CLSM: confocal laser scanning microscopy
